# A Simple Ultrasound Based Classification Algorithm Allows Differentiation of Benign from Malignant Breast Lesions by Using Only Quantitative Parameters

**DOI:** 10.1007/s11307-018-1187-x

**Published:** 2018-04-09

**Authors:** Panagiotis Kapetas, Ramona Woitek, Paola Clauser, Maria Bernathova, Katja Pinker, Thomas H. Helbich, Pascal A. Baltzer

**Affiliations:** 1 Department of Biomedical Imaging and Image-guided Therapy, Medical University of Vienna, Waehringer Guertel 18-20, 1090 Vienna, Austria; 2 Department of Radiology, University of Cambridge, Cambridge Biomedical Campus, Cambridge, CB2 0QQ UK; 3 Molecular Imaging and Therapy Service, Memorial Sloan-Kettering Cancer Center, 301 E 55th St, New York, NY 10022 USA

**Keywords:** Breast cancer, Ultrasound, Doppler ultrasonography, Elastography, Biopsy, Imaging biomarkers, Decision tree

## Abstract

**Purpose:**

We hypothesized that different quantitative ultrasound (US) parameters may be used as complementary diagnostic criteria and aimed to develop a simple classification algorithm to distinguish benign from malignant breast lesions and aid in the decision to perform biopsy or not.

**Procedures:**

One hundred twenty-four patients, each with one biopsy-proven, sonographically evident breast lesion, were included in this prospective, IRB-approved study. Each lesion was examined with B-mode US, Color/Power Doppler US and elastography (Acoustic Radiation Force Impulse–ARFI). Different quantitative parameters were recorded for each technique, including pulsatility (PI) and resistive Index (RI) for Doppler US and lesion maximum, intermediate, and minimum shear wave velocity (SWV_max_, SWV_interm_, and SWV_min_) as well as lesion-to-fat SWV ratio for ARFI. Receiver operating characteristic curve (ROC) analysis was used to evaluate the diagnostic performance of each quantitative parameter. Classification analysis was performed using the exhaustive chi-squared automatic interaction detection method. Results include the probability for malignancy for every descriptor combination in the classification algorithm.

**Results:**

Sixty-five lesions were malignant and 59 benign. Out of all quantitative indices, maximum SWV (SWV_max_), and RI were included in the classification algorithm, which showed a depth of three ramifications (SWV_max_ ≤ or > 3.16; if SWV_max_ ≤ 3.16 then RI ≤ 0.66, 0.66–0.77 or > 0.77; if RI ≤ 0.66 then SWV_max_ ≤ or > 2.71). The classification algorithm leads to an AUC of 0.887 (95 % CI 0.818–0.937, *p* < 0.0001), a sensitivity of 98.46 % (95 % CI 91.7–100 %), and a specificity of 61.02 % (95 % CI 47.4–73.5 %). By applying the proposed algorithm, a false-positive biopsy could have been avoided in 61 % of the cases.

**Conclusions:**

A simple classification algorithm incorporating two quantitative US parameters (SWV_max_ and RI) shows a high diagnostic performance, being able to accurately differentiate benign from malignant breast lesions and lower the number of unnecessary breast biopsies in up to 60 % of all cases, avoiding any subjective interpretation bias.

## Introduction

Ultrasound (US) of the breast is an established adjunct to mammography for the detection and characterization of breast lesions. Despite its high sensitivity, breast US suffers from a low specificity, which results in a high number of false positives and a variable accuracy [1–3] by using morphologic criteria as described in the BI-RADS lexicon [4]. In addition, US is highly operator-dependent, with a generally moderate inter-reader agreement [5–7].

In order to raise the specificity of breast US, several complementary techniques have been introduced, including Doppler and elastography. Doppler evaluates tumor vascularity [3] while elastography provides information about the mechanical properties of tissue [8]. Furthermore, both techniques offer quantitative parameters (pulsed Doppler and shear wave elastography or acoustic radiation force impulse–ARFI) [9–11], which have the potential to be used as imaging biomarkers. Imaging biomarkers are parameters that can be objectively and quantitatively measured using imaging techniques, in order to detect or characterize a disease [12].

Classification algorithms aim to aid in clinical decision making by incorporating different criteria in a formalized manner [13]. Such a formalized and thus objective combination of diagnostic features in the context of a multiparametric approach is supposed to improve specificity and reduce inter-reader variability of breast US. Similar algorithms have been introduced for breast magnetic resonance imaging [14] and demonstrated successfully high diagnostic performance and improved inter-reader agreement [15, 16]. The possibility to establish a comparable classification algorithm for breast US, by incorporating only quantitative data acquired from a multiparametric approach, has not been investigated yet. However, quantitative parameters have the potential to raise the specificity of morphological B-mode US, as well as its reproducibility [9–11, 17, 18].

We hypothesized that different quantitative US parameters may be used as complementary diagnostic criteria and aimed to develop a simple classification algorithm to distinguish benign from malignant breast lesions and aid in the decision to perform biopsy or not.

## Materials and Methods

### Patients

Between October 2015 and September 2016, 124 patients (age range 18–82 years, mean age 52 years) were included in this prospective, IRB-approved, cross-sectional study. Informed consent was obtained from all individual participants included in the study. The study has been performed in accordance with the ethical standards as laid down in the 1964 Declaration of Helsinki and its later amendments. The study participants included both symptomatic and women referred to our breast center for a screening-detected abnormality. Only patients with newly diagnosed, US detected BI-RADS 4 or 5 lesions, which will undergo needle biopsy were included. Exclusion criteria were patient age less than 18 years, pregnancy or lactation, and refusal to undergo a histologic workup. In cases of more than one lesion in the same patient, only the most suspicious or the largest one was included in the study.

### Data Acquisition

All examinations were performed with a Siemens Acuson S3000 device (Siemens Medical Solutions, Mountain View, CA, USA) by one out of a pool of three breast radiologists, with at least 2 years of experience in ARFI elastography and at least 3 years in breast imaging and Doppler US. Lesions were initially identified in B-mode using a linear 18 MHz transducer (18L6HD). Lesion size was defined as the maximum lesion diameter at B-mode US.

Color and Power Doppler examinations were performed using the same transducer for the identification of vessels inside or around (at a 2-mm distance) the lesion [17]. For the Doppler examination, a region of interest (ROI) that was large enough to include the whole lesion and a small amount of surrounding tissue was used. In order to facilitate identification of small vessels, minimal compression was applied, filter settings were set as low as possible, and flow settings were also set to low [17, 19]. Color gain was slowly reduced, until background noise disappeared, in order to achieve maximum sensitivity. When vascularity was detected, pulsed Doppler was used to acquire a spectral waveform of the flow in the most prominent arterial vessel of the tumor. The Doppler angle was kept between 0° and 60° and no angle correction was used. Using the machine’s integrated software, pulsatility (PI) and resistive index (RI) for each vessel were calculated. For this calculation, the examiner chose the best cycle from the Doppler waveform and manually placed the cursor at the maximum systolic and minimum diastolic velocities [17, 20].

Subsequently, the transducer was changed to a linear, 9 MHz one (9L4) and ARFI elastography images of each lesion were acquired. For the ARFI examinations, the latest available software (namely Virtual Touch IQ–VTIQ), already installed in the device, was used. A ROI that was large enough to include the whole lesion and the surrounding tissue was drawn and minimal precompression was applied, in order to avoid artificial tissue stiffening [8, 21]. On the acquired, color-coded image, four 2 × 2-mm-sized quantification ROIs were placed: one on the stiffest area of the lesion (as appreciated on the color-coded velocity map), one at an area of intermediate stiffness, and one at a soft lesion area, as well as a further one on the surrounding fatty tissue at the same depth with the lesion (if this could be included in the image), in order to measure the respective shear wave velocity (SWV) [22]. Using the available quality map, the quantification ROIs were placed on areas of high image quality [23], even in cases when due to a large lesion diameter (approaching the footprint of the transducer), the quality of the measurements was low in the periphery of the lesion. To avoid motion artifacts, the patients were asked in some cases to hold their breath for a couple of seconds [8]. Only one measurement was acquired for each lesion, since a large prospective study has demonstrated shear wave elastography to have an almost perfect intra-reader reproducibility [10]. The measurement scale was adjusted in order to acquire valid measurements (maximum measurable velocity 6.5–10 m/s) [22]. Using the acquired SWV values, a ratio of the intralesional-to-fatty tissue SWV was calculated (lesion-to-fat ratio, L/F ratio) [11].

### Histopathological Examination

All patients underwent US-guided biopsy using a 14G biopsy system (BIP-HistoCore®; BIP Medical, Tuerkenfeld, Germany). The results of the histopathological analysis of the biopsy specimen were used as the reference standard for patients with benign lesions as well as for patients with malignant lesions in case they underwent neoadjuvant chemotherapy (NAC). For patients with malignant tumors not undergoing NAC, as well as for patients receiving surgery due to a lesion with uncertain malignant potential, the post-surgical histopathology result was used as the reference standard.

### Statistical Analysis

The study sample size was calculated based on a hypothetical improvement of the area under the curve (AUC) of B-mode US of the breast from 0.800 to 0.900 with a type I error of 5 % and a statistical power of 80 % through the addition of different quantitative parameters. The benign to malignant ratio was assumed at 0.75, since our clinic is an assessment center for breast imaging with a consecutively high number of breast cancer patients. The values of 0.800 and 0.900 were determined as average values considering several breast US studies [3, 8, 18, 22, 24–27].

Statistical calculations were performed using the software SPSS 20 (IBM Corp, Armonk NY, USA) and MedCalc 12 (MedCalc Software bvba, Ostend, Belgium). No predefined cut-off values for the differentiation between benign and malignant lesions were used. Diagnostic accuracy of all quantitative measurements was evaluated using receiver operating characteristics (ROC) curve analysis. Classification analysis was performed using the exhaustive chi-squared automatic interaction detection (CHAID) method that builds a classification tree with ramifications determined by hierarchical database splits based on chi-square test results. The final diagnostic categories or nodes are characterized by a definite probability of malignancy for specific feature combinations. Minimal parent and child node sizes were set to 10 and 5, respectively. A Bonferroni-adjusted alpha error of 5 % was set as the ramification limit. The classification tree robustness was verified by 10-fold cross-validation [14, 28]. The cut-off values for each ramification were automatically calculated by the CHAID algorithm, based on the iterative testing of possible split values by sequential chi-squared tests. Thus, no predefined cut-off values were used for the analysis.

## Results

### Lesion Characteristics

Sixty-five lesions were malignant (52.4 %) and 59 were benign (47.6 %). The median size of all lesions was 13 mm (range 4–55 mm). The median size of the malignant lesions was 13 mm (Q1 9 mm, Q3 20 mm) and that of the benign ones was 13 mm as well (Q1 10 mm, Q3 22 mm). There was no statistically significant difference between the median sizes between benign and malignant lesions (*p* > 0.05). Histopathological diagnoses of all lesions are summarized in Table 1.

**Table 1:** Detailed histopathological diagnoses of all lesions

Malignant lesions	*n*	Benign lesions	*n*
Invasive ductal carcinoma NOS	53 (81.6 %)	Fibroadenoma	24 (40.7 %)
Invasive lobular carcinoma	6 (9.2 %)	Fibroadenomatous hyperplasia	8 (13.6 %)
Ductal carcinoma *in situ*	4 (6.2 %)	Papilloma	5 (8.5 %)
Mucinous carcinoma	1 (1.5 %)	Fibrosis	4 (6.7 %)
Neuroendocrine carcinoma	1 (1.5 %)	Others	18 (30.5 %)

Percentages are given in parentheses. *NOS* not otherwise specified

### Quantitative Parameters

The diagnostic performance of all quantitative parameters is shown in Table 2. Doppler US identified vessels in 92 out of the 124 lesions. RI was significantly higher in malignant lesions as compared to benign ones and demonstrated an area under the ROC curve (AUC) of 0.642 (cutoff 0.64, *p* = 0.016). The discriminatory power of PI did not reach significant levels.

**Table 2:** Diagnostic performance of all acquired quantitative parameters

Index	AUC	*p* value	Cutoff	Sensitivity	95 % CI	Specificity	95 % CI
PI	0.540	0.523	1.90	77.8	62.9–88.8	39.5	25.0–55.6
RI	0.642	0.016	0.64	78.7	64.3–89.3	53.3	37.9–68.3
SWV_max_	0.867	< 0.0001	3.20	81.5	70.0–90.1	88.1	77.1–95.1
SWV_interm_	0.864	< 0.0001	2.58	86.4	75.0–94.0	80.0	67.0–89.6
SWV_min_	0.829	< 0.0001	2.10	87.1	76.1–94.3	73.2	59.7–84.2
L/F ratio	0.873	< 0.0001	2.06	92.1	82.4–97.4	74.1	61.0–84.7

*PI* pulsatility index, *RI* resistive index, *SWV* shear wave velocity, *SWV*_*max*_ maximum SWV, *SWV*_*interm*_ intermediate SWV, *SWV*_*min*_ minimum SWV

Cut-off values of SWV_max_, SWV_interm_, and SWV_min_ are given in m/s. Sensitivity, specificity, and 95 % confidence intervals in %

All elastography quantitative parameters demonstrated significantly higher values in malignant than in benign lesions. As measured by the AUC, L/F ratio showed the highest performance (cutoff 2.06, AUC 0.873, *p* < 0.001) followed by maximum SWV (SWV_max_ cutoff 3.20 m/s, AUC 0.867, *p* > 0.001).

### Classification Algorithm

**Fig. 1:**
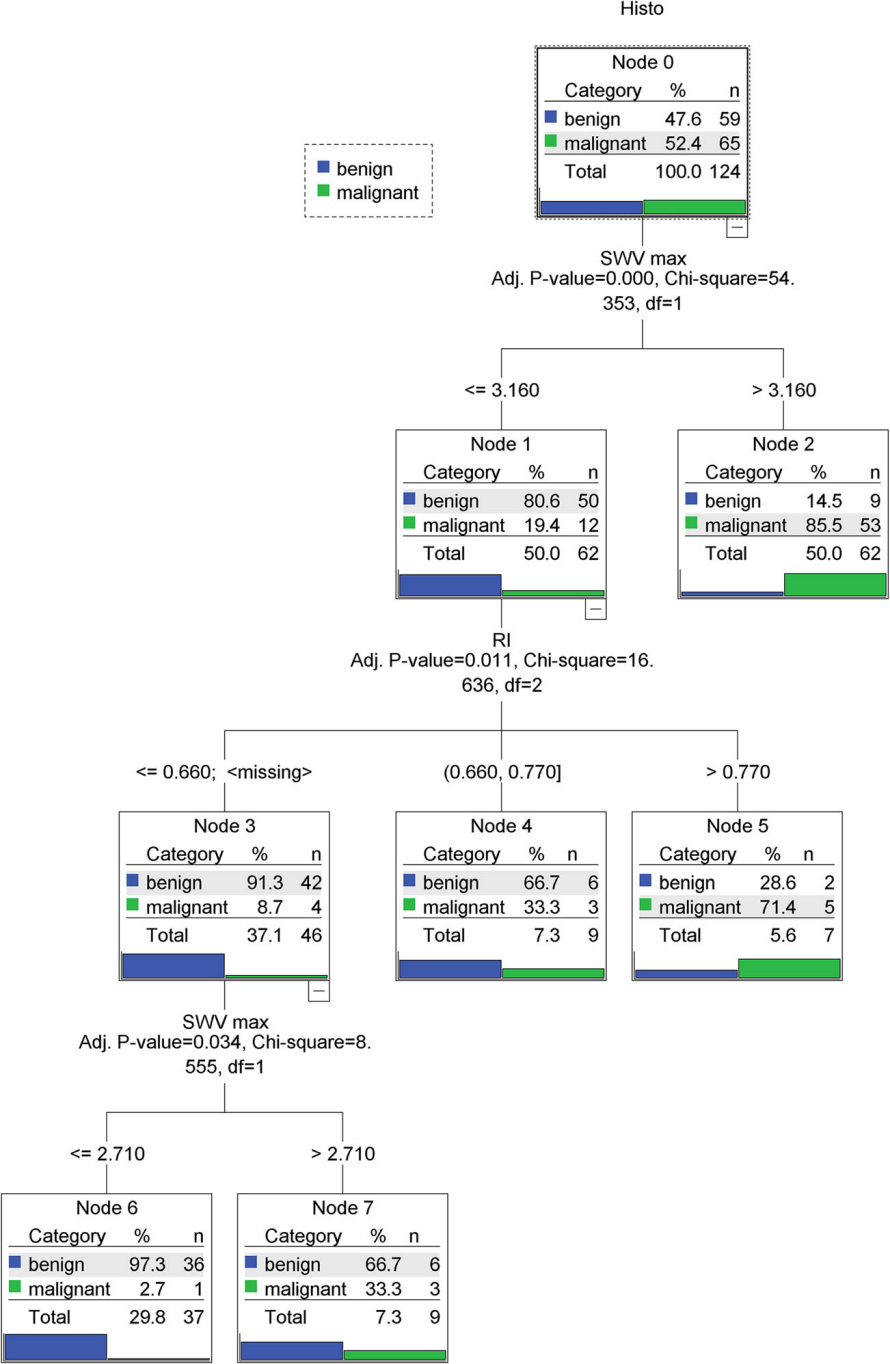
The calculated classification algorithm for the differentiation of benign and malignant lesions. The study population (node 0) is further split into child nodes (1–7), using the variable with the highest discriminating power. After three ramifications, no further discrimination can be achieved. Blue bars demonstrate the percentage of benign and green bars that of malignant lesions in each node. In the first step, SWV_max_ is taken into consideration: if SWV_max_ is higher than 3.16 m/s, the probability of malignancy is > 85 %. If the SWV_max_ is lower than 3.16 m/s, RI is taken into consideration: if the RI is higher than 0.66, the probability of malignancy is still above 33 %. However, in case the RI is lower than 0.66 or no vessels can be detected, SWV_max_ is once again considered. If SWV_max_ is higher than 2.71 m/s, the probability of malignancy surpasses 33 %. In contrast, if SWV_max_ is less than 2.71 m/s, the probability that the lesion is benign is 97.3 %. *RI* resistive index, *SWV*_*max*_ maximum shear wave velocity

The calculated classification algorithm is shown in Fig. 1 and its use is explained in the figure legends. The classification algorithm included two variables, namely SWV_max_ and RI, and showed a depth of three ramifications. All other assessed quantitative parameters did not increase its accuracy and were not included in the algorithm.

In brief, in the first step, SWV_max_ is evaluated: if it is higher than 3.16 m/s, the probability of malignancy is > 85 % and biopsy is warranted. If the SWV_max_ of the lesion is lower than 3.16 m/s, RI is taken into consideration: if this is higher than 0.66, the probability of malignancy is still above 33 %. However, in case the RI is lower than 0.66 or no vessels can be detected in the lesion, SWV_max_ is once again considered. If it is more than 2.71 m/s, the probability of malignancy surpasses 33 %. However, if SWV_max_ is less than 2.71 m/s, the probability that the lesion is malignant falls to 2.7 %, in which case follow-up of the lesion can be safely recommended.

The classification algorithm allowed a risk level assessment with increasing levels of malignancy (node 2 > node 5 > nodes 4 and 7 > node 6) (Table 3). The classification algorithm leads to an AUC of 0.887 (95 % CI 0.818–0.937, *p* < 0.0001), a sensitivity of 98.46 % (95 % CI 91.7–100 %), and a specificity of 61.02 % (95 % CI 47.4–73.5 %).

**Table 3:** Characteristics of the nodes of the classification algorithm

Node	Definition	Predicted category	*n* malignant	*n* benign	*n* total
1	SWV_max_ ≤ 3.16	benign	12 (19.4 %)	50 (80.6 %)	62
*2*	*SWV*_*max*_ *> 3.16*	*malignant*	*53 (85.5 %)*	*9 (14.5 %)*	*62*
3	SWV_max_ ≤ 3.16 and RI ≤ 0.66 or missing	benign	4 (8.7 %)	42 (91.3 %)	46
*4*	*SWV*_*max*_ *≤ 3.16 and RI: 0.66–0.77*	*benign*	*3 (33.3 %)*	*6 (66.7 %)*	*9*
*5*	*SWV*_*max*_ *≤ 3.16 and RI > 0.77*	*malignant*	*5 (71.4 %)*	*2 (28.6 %)*	*7*
*6*	*SWV*_*max*_ *≤ 3.16 and RI ≤ 0.66 or missing and SWV*_*max*_ *≤ 2.71*	*benign*	*1 (2.7 %)*	*36 (97.3 %)*	*37*
*7*	*SWV*_*max*_ *≤ 3.16 and RI ≤ 0.66 or missing and SWV*_*max*_ *> 2.71*	*benign*	*3 (33.3 %)*	*6 (66.7 %)*	*9*

*SWV* shear wave velocity (in m/s), *RI* resistive index

The table refers to the nodes in Fig. 1. Nodes 1 and 3 represent parent nodes, and nodes 2, 4, 5, 6 and 7 (italics) represent terminal nodes. Percentages are given in parentheses

**Fig. 2:**
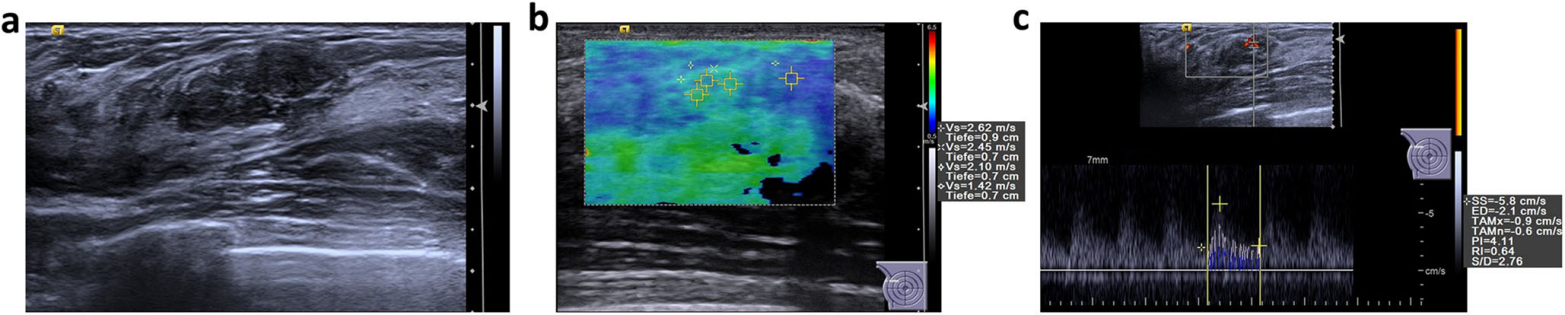
Multiparametric ultrasound of a palpable, 20-mm large lesion in the craniolateral right breast of a 23-year-old patient. **a** In B-mode, the lesion was heterogeneously hypoechoic, lobulated, with a partially indistinct margin and was classified as BI-RADS 4a. **b** In VTIQ elastography, the lesion was overall quite soft, with a SWVmax of 2.62 m/s. **c** Power Doppler detected internal vascularity and the spectrum analysis of the most prominent artery demonstrated an RI of 0.64. The application of the classification algorithm would classify this lesion as node 6, with probability of malignancy of 2.7 %, making a follow-up instead of biopsy a safe alternative. Ultrasound guided biopsy revealed an area of sclerosing adenosis

**Fig. 3:**
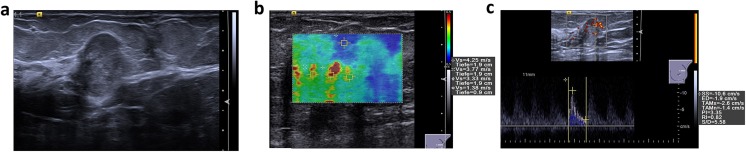
Multiparametric ultrasound of a palpable, 17-mm large lesion in the craniolateral left breast of a 70-year-old patient. **a** In B-mode, the lesion was isoechoic, slightly heterogeneous, with a partially indistinct margin and an anti-parallel orientation and was classified as BI-RADS 4c. **b** In VTIQ elastography, the lesion demonstrated hard parts, with a SWVmax of 4.25 m/s. **c** Power Doppler showed abundant internal vascularity and the spectrum analysis of the most prominent artery demonstrated an RI of 0.82. The application of the classification algorithm would classify this lesion as node 2, with a very high probability of malignancy. Ultrasound guided biopsy revealed a Grade 1 mucinous carcinoma

By applying the proposed algorithm to avoid false positives, 36 out of 59 benign lesions (61 %) could be classified correctly as benign (node 6). Subsequently, an unnecessary biopsy could have been avoided in these 36 cases. The algorithm leads to one false-negative case (2.7 %) (Grade 1 invasive ductal carcinoma) (Figs. 2 and 3).

## Discussion

The results of our study demonstrate that a simple classification algorithm, taking into account two quantitative US parameters (SWV_max_ and RI,) shows a high diagnostic performance, being able to accurately distinguish benign from malignant breast lesions and substantially lower the number of unnecessary breast biopsies.

A significant shortcoming of breast US is its low specificity and positive predictive value, leading to a considerable amount of false-positive results and unnecessary biopsies [1–3]. To overcome this, US techniques other than B-mode have been developed, offering an insight into functional and molecular tissue properties. These include, among others, Doppler US and ARFI elastography, which have shown to raise the specificity of breast US [3, 11, 20]. Our cohort included overall 59 benign lesions and the proposed classification algorithm could correctly classify 36 (61 %) as benign. This in turn means that our algorithm avoids unnecessary biopsy in 36 out of 59 cases.

Any proposed classification algorithm should provide diagnostic certainty in order to be useful in clinical practice. Indeed, our calculated algorithm could provide a definite diagnosis with a diagnostic certainty of more than 97 % for almost 30 % of all cases.

In addition to that, both US techniques that were used can offer quantitative parameters, which provide an objective assessment of tumor vascularity (PI and RI) and stiffness (SWV), thus limiting any subjective interpretation bias. Since the measurements are acquired by the examiner, the presence of measurement and reader bias cannot be excluded. However, several studies have shown that quantitative Doppler US and ARFI elastography are reproducible techniques, with an acceptable intra- and inter-reader agreement [9, 11, 18, 29]. Still, the thresholds determined in the present exploratory study need to be confirmed and eventually adjusted to the respective center and clinical situation they are used in.

Even though it shows a high diagnostic power, one could question why such an algorithm is helpful. Its main advantage, in comparison to B-mode, is the use of quantitative parameters, which may contribute to an increased specificity and reproducibility, allowing for a high diagnostic certainty of the examiner. Obviously, this algorithm aids in the characterization of lesions, assessed at B-mode US.

A lesion classified in the terminal nodes 2, 4, 5, and 7 has a possibility of malignancy of at least 33.3 %. Consequently, a clinical decision to perform biopsy is appropriate. However, lesions classified in the terminal node 6 (SWV_max_ ≤ 2.71 m/s and RI ≤ 0.66 or no vessels detectable) demonstrate a probability of malignancy of 2.7 %. This is only minimally higher than the cutoff for the BI-RADS 3 category (2 %) [4]. Therefore, a short-term follow-up for such lesions seems appropriate, without the risk of missing a significant number of breast cancers.

In our study, several quantitative parameters were evaluated and two of them, namely RI and SWV_max_, were incorporated into the calculated classification algorithm, leading to its high AUC. Several studies have shown that malignant breast lesions demonstrate higher RI values than benign ones. This is usually attributed to the different structure of tumor vessels as compared to normal ones [20, 30–32]. However, other studies demonstrated a significant overlap in RI values between benign and malignant breast lesions, making evident that RI alone has a limited role in their differentiation [17, 33, 34]. On the other hand, shear wave elastography utilizing ARFI imaging has also proven to be useful to distinguish benign from malignant breast lesions [11, 18, 35]. ARFI provides a quantitative measure of tissue stiffness, namely the velocity of shear wave-induced tissue displacement (SWV) [23, 36]. SWV is higher in stiffer (usually malignant) tissues. According to a systematic review by Liu, et al., maximum elasticity in a 2-mm ROI at the stiffest area of the lesion could be the most valuable parameter, which is in line to our findings [27]. Our results support the possibility of utilizing these quantitative parameters as imaging biomarkers, for the differentiation of benign from malignant breast lesions. Although L/F ratio demonstrated a slightly higher AUC than SWV_max_, it was not included into the algorithm by the CHAID. In our clinical experience, SWV_max_ is more reliable than L/F ratio (one measurement less) and thus probably better suited as a predictor for malignancy in clinical practice. This higher reliability of SWV_max_ as compared to L/F ratio has been demonstrated in [11].

According to a meta-analysis by Liu, et al. in 2016 [27], quantitative shear wave elastography shows a pooled sensitivity and specificity of 89 and 87 % respectively. In our study, SWV_max_ also demonstrated a specificity higher than the classification algorithm (88.1 *vs.* 61.1 %). However, both sensitivity and AUC were higher when SWV_max_ was combined with RI than on its own. The high specificity of SWV_max_ came at a cost of a sensitivity of 81.5 %, thus limiting its usefulness in clinical practice. In order for a diagnostic test to be useful in clinical routine, a high sensitivity is mandatory, so as not to miss any significant number of cancers. Our aim was to establish a classification algorithm to aid clinical decision making regarding whether a biopsy should be performed or not. As the priority in trying to omit unnecessary biopsies is not to miss cancer, maintaining a high sensitivity (in our case 98.46 %) is mandatory at the cost of a somewhat lower specificity. This specificity however can be directly translated into the potential to omit unnecessary biopsies which is evidenced by the fact that the specificity of the proposed classification algorithm remains substantially higher than the usually reported specificity of B-mode breast US [3, 37].

Since in our study the elastogram was only acquired in the image with the largest lesion diameter, the possible effects of anisotropy on the measured velocities were not investigated. However, a previous study has demonstrated that anisotropy as such is not plane related [38]. In this study, the number of both benign and malignant lesions demonstrating higher SWVs in the radial plane was almost equal to the ones with higher SWVs in the antiradial plane. Since our images were acquired irrespective of the radial or antiradial plane and the included lesions grew in all different planes, it may be assumed that the effects of anisotropy were averaged, when taking into consideration the whole patient cohort.

Our study had some limitations. First, our study population included a high percentage of malignant lesions (52.4 % of all patients), possibly leading to some degree of spectrum bias. The reason is that it stemmed from an assessment center, where a higher than in the average population pretest probability of malignancy is to be expected. Moreover, we chose to include only cases with a histopathological verification. This aimed at providing a robust standard of reference; however, it may also introduce a sampling bias, since cases with a more straightforward benign diagnosis were excluded from our analysis. Finally, this is a monocentric study with a relatively low number of patients. Although the examinations were performed by different radiologists, trained in different hospitals and with varying levels of experience, our data still need to be tested in a larger validation study, with more participants and examiners. It would be of highest clinical relevance to apply our classification algorithm in a larger number of BI-RADS 3 and 4 lesions to validate its potential to reduce the number of unnecessary benign breast biopsies.

## Conclusions

In conclusion, a simple classification algorithm incorporating two quantitative US parameters (SWV_max_ and RI) shows a high diagnostic performance, being able to accurately differentiate benign from malignant breast lesions and lower the number of unnecessary breast biopsies in up to 60 % of all cases, avoiding any subjective interpretation bias.
